# Machine learning model for unfavorable outcome prediction in neurosurgical patients: the potential role of liver function markers

**DOI:** 10.3389/fneur.2026.1779349

**Published:** 2026-04-29

**Authors:** Yibo Fan, Jingyue Zhang, Lin Wu, Shuo An, Yingsheng Wei, Jian Sun, Ye Tian, Hanxu Zhang

**Affiliations:** 1 Department of Neurosurgery, Tianjin Medical University General Hospital, Tianjin, China; 2 Department of Pharmacy, Tianjin Medical University General Hospital, Tianjin, China

**Keywords:** liver function markers, machine learning, neurocritical care, neurosurgery, prognostic prediction

## Abstract

**Introduction:**

Early prediction of prognosis for neurosurgical diseases remains challenging. This study aimed to develop a machine learning-based model to predict unfavorable outcomes in neurosurgical patients.

**Methods:**

We conducted a retrospective cohort study of patients with traumatic brain injury, intracerebral hemorrhage, or aneurysmal subarachnoid hemorrhage between 2018 and 2020. The primary outcome was functional status at discharge, assessed via the modified Rankin Scale. Feature selection used LASSO regression and the Boruta algorithm, with overlapping selected features retained for model development. Six machine learning algorithms were trained with 5-fold cross-validation for hyperparameter optimization via Optuna. Model performance was evaluated using area under the receiver operating characteristic curve (AUC), calibration curves, and decision curve analysis. Shapley additive explanations were used for interpretability.

**Results:**

The CatBoost model performed best (AUC = 0.932, accuracy = 0.879, precision = 0.872, recall = 0.810, F1 score = 0.840, Brier score = 0.116), balancing discriminative power and clinical relevance. Key predictive features included Glasgow Coma Scale (GCS) score at admission, age, and liver function markers including aspartate transaminase (AST) _mean_, albumin _mean_, alkaline phosphatase (ALKP) _mean_, ALKP _max_, albumin _min_, and ALKP _first_. Lower GCS score at admission and older age predicted unfavorable outcomes. Higher mean AST, mean ALKP and initial ALKP, as well as lower mean and minimum albumin, were associated with unfavorable outcomes.

**Discussion:**

The CatBoost model showed excellent performance in predicting the prognosis of neurosurgical patients by integrating neurological and liver function markers. Future studies are needed for external validation through multicenter investigations, and explore mechanistic associations between liver dysfunction and neurological deterioration.

## 1 Introduction

Neurosurgical diseases, including traumatic brain injury (TBI) and spontaneous intracerebral hemorrhage (ICH), exhibit a high incidence rate and represent one of the leading causes of death and long-term disability worldwide (1, 2). It is well recognized that establishing reliable prognostic assessments in the early phase after TBI remains a notable challenge, while ICH patients face a relatively high 30-day mortality rate (≤40%) (3), with two-thirds of survivors experiencing moderate to severe disability (4, 5). Early identification of patients at risk for poor prognosis plays a critical role in enabling timely interventions, refining treatment strategies, and improving clinical outcomes.

In recent years, the influence of systemic organ dysfunction on neurosurgical prognosis has garnered increasing attention, with the bidirectional interaction between hepatic and neurological function, the “liver-brain axis,” emerging as a focal point of research (6). Hepatic dysfunction impairs neurological function through multiple interconnected mechanisms: urea cycle impairment leads to hyperammonemia, triggering astrocyte swelling and cerebral edema; cholestasis induces manganese deposition in the basal ganglia, contributing to parkinsonism-like symptoms; and intestinal barrier disruption with endotoxin translocation activates neuroinflammatory pathways, exacerbating blood–brain barrier damage (7).

Clinical studies have confirmed that liver function markers, including elevated alanine transaminase (ALT)/aspartate transaminase (AST) (8), reduced albumin-to-alkaline phosphatase ratio (AAPR) (9), and elevated serum alkaline phosphatase (ALKP) (10), serve as risk factors for neurological deterioration and increased mortality in neurosurgical patients. Additionally, serum albumin, a key hepatic synthetic protein with antioxidant, anti-inflammatory, and neuroprotective properties, has demonstrated potential in predicting poor outcomes across a spectrum of neurosurgical conditions (11, 12).

Despite growing recognition of the liver-brain axis’ clinical significance, comprehensive evaluation of liver function markers as prognostic indicators across diverse neurosurgical populations remains limited. Current research predominantly focuses on specific subtypes such as ischemic stroke or subarachnoid hemorrhage, with insufficient investigation into TBI, ICH, and other neurosurgical conditions. Traditional statistical models also fail to capture non-linear relationships and complex interactions between liver function markers and neurological outcomes, resulting in underutilization of their potential prognostic value. In predicting outcomes for patients undergoing cerebrovascular or neuroendovascular surgery, machine learning could outperform traditional clinical and statistical models (13).

The present study aims to construct a machine learning-based predictive model for adverse prognosis in neurosurgical patients, with a primary focus on investigating the predictive value of liver function markers in the model and their enhancing effect on model performance.

## 2 Methods

### 2.1 Study population

This retrospective cohort study included all consecutive patients (≥16 years) with specific diagnoses of TBI, ICH, or aneurysmal subarachnoid hemorrhage (aSAH) in the Department of Neurosurgery, Tianjin Medical University General Hospital, China between January 2018 and December 2020. Inclusion criteria were: (1) primary diagnosis of TBI, ICH, or aSAH requiring hospitalization, (2) availability of liver function tests during admission, (3) documented functional outcome at discharge using modified Rankin Scale (mRS). Exclusion criteria included: (1) pre-existing severe liver disease (cirrhosis, hepatitis B or C infection, autoimmune hepatitis), (2) concurrent hepatotoxic medication use prior to admission, (3) alcohol use disorder, (4) malignancy with known hepatic involvement, (5) incomplete medical records, and (6) discharge within 24 h of admission. This study was approved by the ethics committees of Tianjin Medical University General Hospital (approval number: IRB2020-DW-19), and the requirement for informed consent was waived due to the retrospective nature of the investigation.

### 2.2 Data collection and variables

Patient demographics, medical history, and clinical characteristics were extracted from electronic medical records. Variables included age, sex, primary neurosurgical diagnosis (TBI, ICH and aSAH), comorbidites, surgical intervention status, intensive care unit (ICU) admission, and Glasgow Coma Scale (GCS) score at admission. For patients with multiple concurrent diagnoses, the primary admission diagnosis determining the need for hospitalization was used for classification. Traumatic subarachnoid hemorrhage was classified under TBI rather than SAH. Liver function test data were collected prior to functional outcome assessment at discharge, including ALT, AST, ALKP, gamma-glutamyl transferase (GGT), lactate dehydrogenase (LDH), total bilirubin (TBil), albumin, globulin, and total protein. For each indicator, we extracted three features: the first recorded value (baseline), the mean (reflecting sustained burden), and the maximum or minimum value to capture acute disturbance. Specifically, maximum values were used for injury or cholestasis markers (GGT, ALT, AST, ALKP, LDH, TBil), and minimum values for synthetic markers (albumin, globulin, total protein). This multi-dimensional approach enables the model to evaluate the association between initial status, peak injury, sustained dysfunction, and neurological outcome. The primary outcome was functional status at hospital discharge, assessed using the mRS. Outcomes were dichotomized as favorable (mRS 0–2) versus unfavorable (mRS 3–6).

### 2.3 Data preprocessing

Isolation forest was used for outlier detection, and variables with outliers were transformed into logarithmic space to avoid the effect of outliers. For missing data, variables with more than 20% missing values were removed to minimize possible bias due to imputation. And the Multivariate Imputation Chained Equations (MICE) (14) was performed to impute the remaining variables. The missing data status for all collected variables is detailed in Supplementary Table 1. The data were normalized to prevent the model from being biased by differences in feature scales.

### 2.4 Feature selection

The Least Absolute Shrinkage and Selection Operator (LASSO) regression (15) and the Boruta (16) algorithm were used for feature selection. LASSO regression achieves feature selection by compressing coefficients of unimportant features to zero through L1 regularization. It handles high-dimensional data, avoids overfitting, and provides feature coefficient estimates. The Boruta algorithm evaluates feature importance using random forests, selecting features by comparison with shadow features. It handles high-dimensional data and offers more robust evaluation. The intersection of important features selected by both methods precisely identifies core features deemed important by all approaches. All feature selection procedures were conducted exclusively on the training set to prevent data leakage.

### 2.5 Model development and evaluation

**Figure 1:**
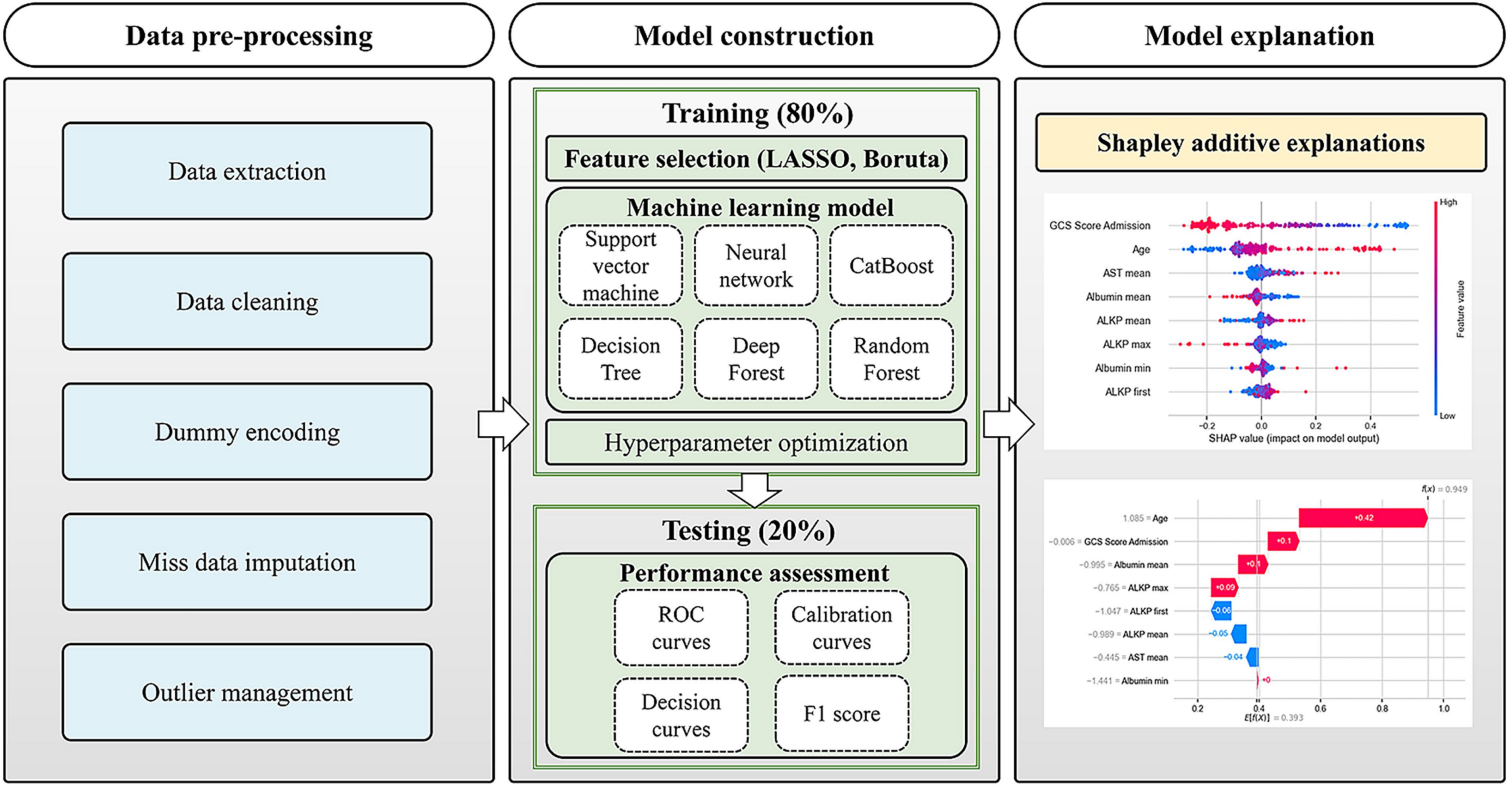
Research process flow diagram.

The dataset was stratified and randomly divided into training and testing sets in an 8:2 ratio. We employed six machine learning algorithms to develop predictive models: Deep Forest, Decision Tree, Support Vector Machine (SVM), Categorical Boosting (CatBoost), Neural Network, and Random Forest. Each model used a 5-fold cross-validation process to optimize the model hyperparameters based on the open source optimization framework Optuna (version 2.10.0) (17). Model performance was evaluated and compared based on metrics including area under the receiver operating characteristic curve (AUC), accuracy, specificity, precision, sensitivity, and F1 score. Additionally, calibration curves were plotted to assess the consistency between predicted probabilities and actual outcomes, and Brier score was used to assess the calibration of the model. To further evaluate the models’ potential clinical utility, decision curve analysis (DCA) (18) was performed to quantify the net benefit across various threshold probabilities. The shapley additive explanations (SHAP) (19) were used to interpret the model at the global and local levels. The research process is illustrated in Figure 1.

### 2.6 Statistical analysis

The analytical approach undertaken in this study included statistical methods and machine learning with hyperparameter tuning, implemented in Python 3.8.3 using scikit-learn. The normality of continuous variables was assessed via the Shapiro–Wilk test. Non-normally distributed variables were presented as median with interquartile range (IQR) and compared using the Mann–Whitney U test. Categorical variables were presented as frequencies with percentages and compared using the chi-square test or Fisher’s exact test when appropriate.

## 3 Results

### 3.1 Clinical characteristics

A total of 1,069 patients met the inclusion criteria and were included in the final analysis. The cohort consisted of 681 males (63.7%) and 388 females (36.3%), with a median age of 61 years. The primary diagnoses were distributed as follows: TBI identified in 413 patients (38.6%), ICH in 490 patients (45.8%), and aSAH in 166 patients (15.5%). At hospital discharge, 649 patients (60.7%) achieved favorable outcomes (mRS 0–2), while 420 patients (39.3%) had unfavorable outcomes (mRS 3–6). Baseline characteristics and liver function parameters are presented in Table 1 and Supplementary Table 2, respectively.

**Table 1:** Baseline characteristics of the study population.

Variables	Total (*n* = 1,069)	Favorable outcome (*n* = 649)	Unfavorable outcome (*n* = 420)	*p*
Demographics				
Male sex, *n* (%)	681 (63.7)	419 (64.6)	262 (62.4)	0.51
Age, median (IQR)	61.0 (49.0,70.0)	56.0 (46.0,66.0)	67.0 (56.0, 77.0)	<0.001
Disease type, *n* (%)				<0.001
TBI	413 (38.6)	317 (48.8)	96 (22.9)	
ICH	490 (45.8)	205 (31.6)	285 (67.9)	
SAH	166 (15.5)	127 (19.6)	39 (9.3)	
Treatment variables				
Surgical intervention, *n* (%)	426 (39.9)	224 (34.5)	202 (48.1)	<0.001
ICU admission, *n* (%)	805 (75.3)	417 (64.3)	388 (92.4)	<0.001
GCS admit category, *n* (%)				<0.001
Mild (13–15)	625 (58.5)	499 (76.9)	126 (30.0)	
Moderate (9–12)	225 (21.0)	104 (16.0)	121 (28.8)	
Severe (3–8)	219 (20.5)	46 (7.1)	173 (41.2)	
Comorbidites				
Hypertension, *n* (%)	556 (52.0)	330 (50.8)	226 (53.8)	0.377
Diabetes, *n* (%)	173 (16.2)	94 (14.5)	79 (18.8)	0.073
CHD, *n* (%)	136 (12.7)	71 (10.9)	65 (15.5)	0.038
Stroke/Hemorrhage, *n* (%)	188 (17.6)	87 (13.4)	101 (24.0)	<0.001

CHD, coronary heart disease; GCS, Glasgow Coma Scale; ICH, intracerebral hemorrhage; ICU, intensive care unit; IQR, interquartile range; SAH, subarachnoid hemorrhage; TBI, traumatic brain injury.

### 3.2 Feature selection

**Figure 2:**
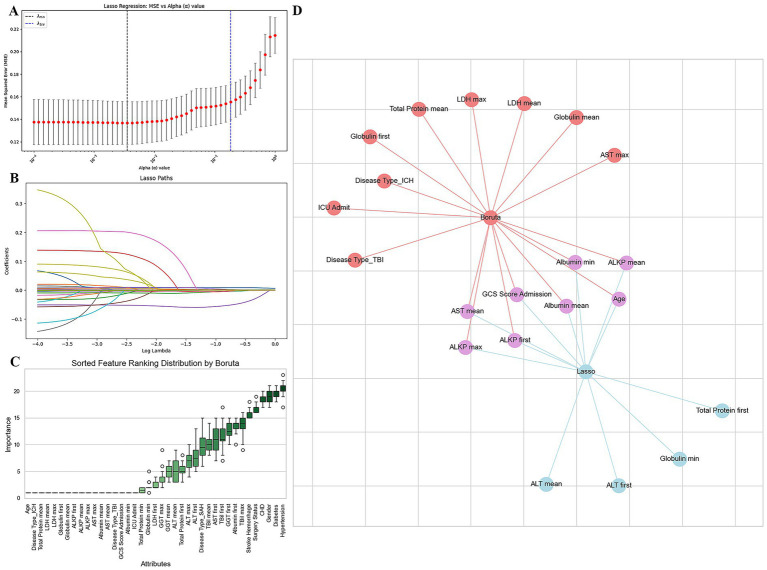
Predictor screening by LASSO and Boruta. **(A)** LASSO regression model factor selection; **(B)** LASSO regression model screening variable trajectories; **(C)** Boruta factor selection; **(D)** Variables identified using the intersection of the LASSO and Boruta algorithm.

The most important variables were identified using the intersection of the LASSO and Boruta algorithm (Figure 2). Eight most significant features were determined and used as input variables for subsequent prediction model construction, including GCS score at admission, age, AST _mean_, albumin _mean_, ALKP _mean_, ALKP _max_, albumin _min_, and ALKP _first_.

### 3.3 Model construction and performance

**Figure 3:**
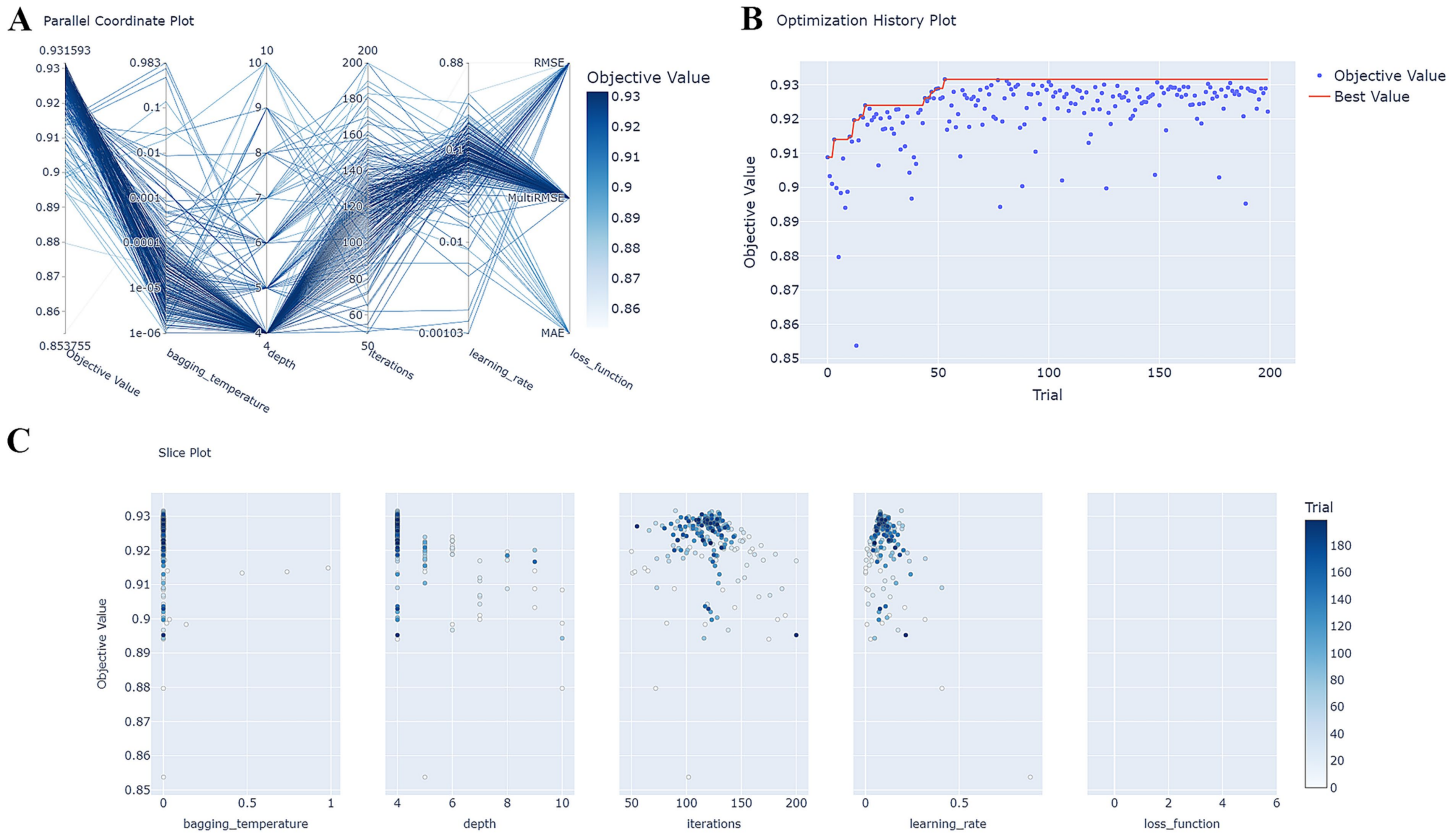
HPO process for the CatBoost model. **(A)** Parallel coordinate system plot showing the distribution of hyperparameters in the CatBoost model during HPO, where dark colors are matched with higher AUC values; **(B)** Optimization history plot showing the trajectory of the evolution of the optimal values with the number of training sessions during the HPO process; **(C)** Slice plot shows the correlation between each hyperparameter and AUC. AUC, area under the receiver operating characteristic curve; HPO, hyperparameter optimization.

Hyperparameter optimization (HPO) was conducted for six models, with optimal hyperparameter combinations yielding the highest cross-validation AUC scores determined following 200 trials. The HPO process for the CatBoost model is illustrated in Figure 3. The CatBoost model was ultimately trained using the following hyperparameter settings: loss_function = MultiRMSE, iterations = 90, learning_rate = 0.1921860357535433, depth = 4, bagging_temperature = 6.994238969249277e-06. The final hyperparameters for the remaining five models are presented in Supplementary Table 3.

**Figure 4:**
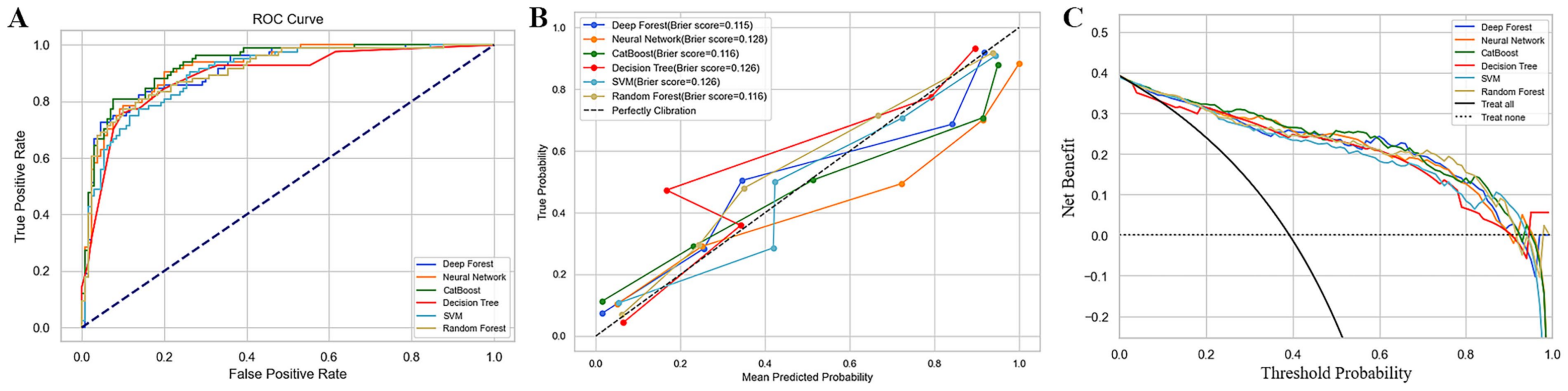
Performance of the six machine learning models: **(A)** Receiver operating characteristic curves; **(B)** Calibration curves; **(C)** Decision curve analysis.

Based on optimal hyperparameters, six models were developed using training data and evaluated on the test set. The CatBoost model demonstrated superior discriminative performance with an AUC of 0.932 (Figure 4A), an accuracy of 0.879, and a precision of 0.872, outperforming other models (Table 2). The F1 score (0.840) further confirmed CatBoost’s balance of precision (0.872) and recall (0.810), surpassing other models. Regarding probabilistic calibration, CatBoost also yielded a low Brier score of 0.116 (Figure 4B). DCA further validated CatBoost’s clinical utility, showing a higher net benefit across threshold probabilities (Figure 4C). Collectively, these results indicate CatBoost as the most effective model for the current task, balancing discriminative power, class-specific performance, and clinical relevance.

**Table 2:** Computational performance of CatBoost as compared to other models.

Model	Accuracy	Precision	Recall	F1	AUC	Brier
Deep forest	0.846	0.793	0.821	0.807	0.912	0.115
Neural network	0.841	0.745	0.905	0.817	0.921	0.128
CatBoost	0.879	0.872	0.810	0.840	0.932	0.116
Decision tree	0.846	0.831	0.762	0.795	0.890	0.126
SVM	0.804	0.691	0.905	0.784	0.900	0.126
Random forest	0.850	0.833	0.774	0.802	0.908	0.116

AUC, receiver operating characteristic curve; CatBoost, categorical boosting; SVM, support vector machine.

### 3.4 Model interpretation

**Figure 5:**
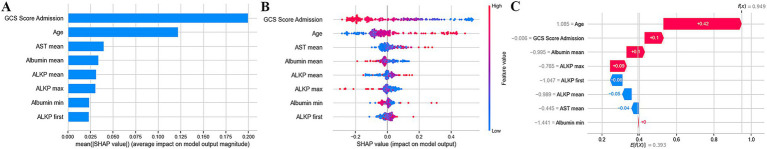
Matrix plots of features. **(A)** A bar plot illustrates the feature importance values determined by SHAP, where a higher value indicates a greater contribution to the model’s predictions; **(B)** A violin plot depicts distribution of SHAP summary values for each feature across all samples; **(C)** Individual prediction interpretation of the CatBoost model based on the SHAP method. SHAP, Shapley additive explanations.

The SHAP analysis of the CatBoost model provided insights into the ranking of key features (Figure 5A) and the overall correlation and directionality between features and outcomes (Figure 5B). The top eight most important features identified were: GCS score at admission, age, AST _mean_, albumin _mean_, ALKP _mean_, ALKP _max_, albumin _min_, and ALKP _first_. A lower GCS score at admission and older age predict an unfavorable outcome. Notably, the study revealed associations between multiple liver function markers and prognosis. A higher average AST level during hospitalization was associated with unfavorable outcomes. Additionally, lower average and minimum albumin levels were associated with an elevated risk of unfavorable prognosis. ALKP levels during hospitalization were also found to be related to functional prognosis. Figure 5C presents the individual prediction explanations for the CatBoost model, obtained using the SHAP method.

To further evaluate the value of liver function markers in optimizing the model, we retained only the two most influential predictors for functional prognosis, GCS score and age, to re-built the machine learning prediction model. The results showed that the Catboost model still performed the best, with an AUC of 0.922 and an accuracy of 0.832. However, the overall prediction effect of this model was inferior to that of the model including liver function markers (Supplementary Table 4). The hyperparameters are presented in Supplementary Table 5.

## 4 Discussion

Predicting the prognosis of neurosurgical patients holds significant clinical importance, as it serves to guide the optimization of treatment strategies and nursing intensity. In resource-limited settings, the model prioritizes patients most likely to benefit from critical care resources, optimizing healthcare efficiency without compromising outcomes. This study developed a machine learning-based prognostic prediction model for neurosurgical patients. Among the six algorithms evaluated, the CatBoost model achieved the highest predictive efficacy, with an AUC of 0.932, accuracy of 0.879, and precision of 0.872, outperforming other models. The SHAP analysis identified admission GCS score and age as the most influential predictors of adverse prognosis, aligning with established evidence (20–22). Notably, liver function markers (AST, albumin, ALKP) emerged as key contributors. Elevated levels of AST _mean_, ALKP _mean_, and ALKP _first_, along with reduced levels of albumin _mean_ and albumin _min_, were associated with higher SHAP values for unfavorable outcomes. These findings provide empirical support for the “liver-brain axis” hypothesis, highlighting the bidirectional interplay between hepatic dysfunction and neurological recovery.

Machine learning has demonstrated excellent performance in outcome prediction for a wide range of neurosurgical conditions (23), and its predictive performance is generally superior to that of traditional statistical models and clinical scoring systems (13). A systematic review and meta-analysis showed that in the prediction of mRS functional outcomes after mechanical thrombectomy, the AUC of the pooled test set reached 0.84 (95%CI 0.79–0.88). For the prediction of functional outcomes in SAH patients, the pooled AUC was higher (0.89, 95%CI 0.76–0.95) (13). While machine learning studies on TBI using mRS as the functional outcome remain limited, such models have demonstrated high accuracy in predicting poor functional prognosis in TBI patients via GCS, with accuracy generally exceeding 80% (24).

Compared with previous studies, the present study has several strengths. First, the variables incorporated into the model are laboratory indicators widely used in clinical practice, which are routinely measured during hospitalization without involving high costs or technical challenges. This ensures the model’s predictive accuracy while facilitating its practical implementation. Second, this study included patients with three common neurosurgical diseases, and the diverse disease spectrum enhances its suitability for clinical application in neurosurgery. Moreover, our model elucidated the contributions of influencing factors through SHAP-based interpretability analysis. In summary, the development of this predictive model may assist in clinical decision-making for initial treatment and guide family discussions and counseling.

Our previous study demonstrated that TBI exacerbates drug-induced liver injury via oxidative stress, inflammation, and apoptosis (25), providing a mechanistic basis for the prognostic value of liver function markers identified in the current study. The present study identified serum albumin level as a significant predictor of prognosis in neurosurgical patients, with lower levels indicating a higher risk of unfavorable outcomes. This finding is highly consistent with previous studies on neurocritical diseases including aSAH, TBI, ICH, and ischemic stroke (11, 12, 26, 27). Synthesized by hepatocytes, albumin is not only a critical negative acute-phase protein, but also has inherent antioxidant activity. It exerts neuroprotective effects through multiple mechanisms, including antioxidation, anti-inflammation, antithrombosis, and the maintenance of plasma colloid osmotic pressure (28–31). Preclinical animal studies have demonstrated that exogenous human albumin supplementation in aSAH and TBI models can improve neurological deficits, reduce lesion volume, and alleviate long-term neurobehavioral sequelae (31–33). Clinical studies have also preliminarily verified the safety of albumin supplementation and its potential to improve patient outcomes (28). Therefore, a low albumin level at admission not only reflects exacerbated systemic inflammatory response and malnutrition, but may also indicate impaired endogenous neuroprotective mechanisms, thereby correlating with an increased risk of adverse clinical events such as delayed cerebral ischemia (11), post-traumatic cerebral infarction (12), and poor functional prognosis. Furthermore, the predictive value of albumin is also reflected in its derived composite indicators, including the albumin-to-fibrinogen ratio (AFR) (34), C-reactive protein-to-albumin ratio (CAR) (35), neutrophil-to-albumin ratio (NAR) (36–40), and the HALP (hemoglobin, albumin, lymphocyte, and platelet) score (41), etc. This further supports that a multidimensional assessment integrating inflammatory and nutritional status can reflect disease severity more comprehensively. Our study incorporates albumin, a readily available routine clinical indicator, into the predictive model, reinforcing its practical value in early risk stratification of critically ill neurosurgical patients. It also provides indirect theoretical support for hypoalbuminemia-targeted interventions, such as nutritional support or albumin supplementation, to improve patient prognosis. Future large-scale prospective studies are warranted to validate whether dynamic monitoring of albumin or its related composite indicators can provide more precise guidance for prognostic assessment.

ALKP is a membrane-bound metalloenzyme that catalyzes phosphate ester hydrolysis. Clinically, serum ALKP is commonly utilized as a diagnostic marker for liver disease or biliary obstructive disease (10). In patients with acute ischemic stroke, serum ALKP levels are independently associated with 3-month poor prognosis (mRS > 2), with a 1.21-fold increased risk observed when ALKP ≥ 288 U/L (10). In the context of ICH, ALKP levels exhibit a positive correlation with hematoma volume and a negative correlation with GCS scores. A study demonstrated that serum ALKP levels >78.5 U/L predicted unfavorable prognosis with 69.1% sensitivity and 83.6% specificity (42). Another study showed that in patients with spontaneous ICH, a high ALKP level (>94.8 U/L) was identified as an independent predictor of poor functional outcomes at 30 days, 90 days, and 1 year (43). Mechanistically, ALKP may play a role in blood–brain barrier disruption, neuroinflammation, and vascular dysfunction in stroke patients (44). Elevated ALKP levels might reflect malnutrition or liver dysfunction, thereby indirectly impairing neurorepair capacity. A low albumin-to-alkaline phosphatase ratio indicates a dominant inflammatory response in the patient’s body, accompanied by poor nutritional status, which may contribute to adverse clinical outcomes (9). However, controversies exist regarding the prognostic value of ALKP: some studies have demonstrated no significant association with clinical outcomes (45), potentially attributable to variations in sample size, stroke subtypes, or comorbid patient characteristics. Notably, in the SHAP analysis, higher ALKP _max_ was associated with a lower risk of unfavorable outcomes, which showed an opposite trend compared with ALKP _mean_ and ALKP _first_. Although multicollinearity was partially alleviated by the LASSO and Boruta feature selection procedures, inherent correlations still existed among these derived ALKP parameters. This counterintuitive pattern may reflect the conditional effects captured by the machine learning model rather than a direct causal association.

This study has several limitations. First, its retrospective design carries inherent constraints, including potential data incompleteness and limited representativeness of the study population. As with any observational study, residual confounding from unmeasured or unrecorded variables cannot be entirely excluded, which may affect the validity of the predictor-outcome estimates. Second, functional outcomes were assessed only at discharge using the mRS, without longer-term follow-up. Thus, the current model is more suitable for the pre-discharge context. In subsequent work, we will incorporate follow-up data to build a comprehensive prognostic system covering the full disease course, thereby expanding its scope of application. Third, the model was developed using data from a single tertiary center, which limits its generalizability to other settings due to variations in treatment protocols and patient characteristics. External validation in diverse clinical settings is therefore essential before broader application. To address this limitation, we plan to conduct an external validation using public critical care databases, including MIMIC-IV and eICU, with the same inclusion and exclusion criteria applied in the current study. Fourth, the selection of variables was not fully comprehensive. For instance, basic vital sign data monitored by intensive care devices such as electrocardiographic monitors, ventilators, and pressure sensors were not recorded. The inclusion of these parameters might have offered deeper insights into the patients’ condition. Despite these limitations, this study demonstrates the significant predictive value of commonly available laboratory indicators, such as liver function tests, and supports their use in facilitating more informed clinical decision-making.

## 5 Conclusion

In summary, our CatBoost model demonstrated excellent performance in predicting the prognosis of neurosurgical patients by integrating neurological and liver function indicators. These findings underscore the clinical significance of the liver-brain axis and support the adoption of machine learning tools to optimize patient management. Future studies should validate the model through multicenter investigations, update dynamic models by incorporating longitudinal data to enhance translational potential, and further explore the mechanistic associations between liver dysfunction and neurological deterioration, with a focus on pathways such as neuroinflammation and oxidative stress.

## Data Availability

The raw data supporting the conclusions of this article will be made available by the authors, without undue reservation.
