# Supplementary material for: Machine learning model for unfavorable outcome prediction in neurosurgical patients: the potential role of liver function markers
Source: Front Neurol. 2026 Apr 29;17:1779349. doi: 10.3389/fneur.2026.1779349 (PMC13167422; doi:10.3389/fneur.2026.1779349)
Supplement: Supplementary file 1 [file Table_1.DOCX]

Supplementary Material

# Supplementary Table 1

**Supplementary Table 1.** Missing data description for all variables.

| **Variable** | **Variable type** | **Missing count, n (%)** |
| --- | --- | --- |
| Age | Continuous | 0 (0.00%) |
| Gender | Categorical | 0 (0.00%) |
| Surgical intervention | Categorical | 0 (0.00%) |
| ICU admission | Categorical | 0 (0.00%) |
| GCS score admission | Continuous | 0 (0.00%) |
| Hypertension | Categorical | 0 (0.00%) |
| Diabetes | Categorical | 0 (0.00%) |
| Coronary heart disease | Categorical | 0 (0.00%) |
| Stroke/Hemorrhage | Categorical | 0 (0.00%) |
| ALT _max_ | Continuous | 0 (0.00%) |
| ALT _mean_ | Continuous | 0 (0.00%) |
| ALT _first_ | Continuous | 0 (0.00%) |
| AST _max_ | Continuous | 0 (0.00%) |
| AST _mean_ | Continuous | 0 (0.00%) |
| AST _first_ | Continuous | 0 (0.00%) |
| ALKP _max_ | Continuous | 0 (0.00%) |
| ALKP _mean_ | Continuous | 0 (0.00%) |
| ALKP _first_ | Continuous | 0 (0.00%) |
| GGT _max_ | Continuous | 0 (0.00%) |
| GGT _mean_ | Continuous | 0 (0.00%) |
| GGT _first_ | Continuous | 0 (0.00%) |
| LDH _max_ | Continuous | 0 (0.00%) |
| LDH _mean_ | Continuous | 0 (0.00%) |
| LDH _first_ | Continuous | 0 (0.00%) |
| TBil _max_ | Continuous | 0 (0.00%) |
| TBil _mean_ | Continuous | 0 (0.00%) |
| TBil _first_ | Continuous | 0 (0.00%) |
| Albumin _min_ | Continuous | 5 (0.47%) |
| Albumin _mean_ | Continuous | 11 (1.03%) |
| Albumin _first_ | Continuous | 1 (0.09%) |
| Globulin _min_ | Continuous | 0 (0.00%) |
| Globulin _mean_ | Continuous | 0 (0.00%) |
| Globulin _first_ | Continuous | 0 (0.00%) |
| Total protein _min_ | Continuous | 0 (0.00%) |
| Total protein _mean_ | Continuous | 0 (0.00%) |
| Total protein _first_ | Continuous | 0 (0.00%) |
| Disease type | Categorical | 0 (0.00%) |

ALKP, alkaline phosphatase; ALT, alanine transaminase; AST, aspartate transaminase; CHD, coronary heart disease; GCS, Glasgow Coma Scale; GGT, gamma-glutamyl transferase; ICU, intensive care unit; LDH, lactate dehydrogenase; TBil, total bilirubin.

# Supplementary Table 2

**Supplementary Table 2.** Liver function parameters in the study population.

| **Variables** | **Total (n = 1069)** | **Favorable Outcome (n=649)** | | **Unfavorable Outcome (n=420)** | | **P** |
| --- | --- | --- | --- | --- | --- | --- |
| **GGT** |  | |  | |  |  |
| max, median (IQR) | 49.0 (27.0, 121.0) | | 44.0 (25.0, 94.0) | | 62.0 (31.0, 144.0) | <0.001 |
| mean, median (IQR) | 34.5 (21.0, 71.2) | | 32.3 (19.8, 62.0) | | 39.0 (23.1, 82.3) | <0.001 |
| first, median (IQR) | 23.0 (16.0, 39.0) | | 23.0 (15.0, 39.0) | | 24.0 (16.0, 39.0) | 0.488 |
| **Albumin** |  | |  | |  |  |
| min, median (IQR) | 31.0 (27.0, 36.0) | | 34.0 (29.0, 37.0) | | 28.0 (25.0, 32.0) | <0.001 |
| mean, median (IQR) | 36.2 (32.8, 39.3) | | 37.7 (34.7, 40.6) | | 33.8 (31.1, 37.2) | <0.001 |
| first, median (IQR) | 43.0 (39.0, 46.0) | | 43.0 (39.0, 46.0) | | 42.0 (39.0, 46.0) | 0.524 |
| **ALT** |  | |  | |  |  |
| max, median (IQR) | 51.0 (30.0, 96.0) | | 46.0 (29.0, 87.0) | | 60.0 (33.0, 109.2) | <0.001 |
| mean, median (IQR) | 34.3 (22.5, 52.5) | | 33.0 (21.7, 49.2) | | 36.9 (24.5, 59.8) | 0.001 |
| first, median (IQR) | 27.0 (21.0, 37.0) | | 27.0 (21.0, 37.0) | | 27.0 (20.0, 37.0) | 0.433 |
| **AST** |  | |  | |  |  |
| max, median (IQR) | 52.0 (33.0, 102.0) | | 44.0 (31.0, 79.0) | | 68.0 (39.8, 129.0) | <0.001 |
| mean, median (IQR) | 35.0 (24.6, 57.0) | | 31.3 (23.2, 47.5) | | 44.5 (28.1, 70.4) | <0.001 |
| first, median (IQR) | 32.0 (25.0, 43.0) | | 31.0 (25.0, 42.0) | | 33.0 (25.0, 46.0) | 0.046 |
| **ALKP** |  | |  | |  |  |
| max, median (IQR) | 101.0 (78.0, 137.0) | | 93.0 (74.0, 126.0) | | 111.0 (86.0, 152.0) | <0.001 |
| mean, median (IQR) | 80.7 (64.5, 102.5) | | 76.5 (61.5, 96.5) | | 87.2 (69.9, 108.8) | <0.001 |
| first, median (IQR) | 81.0 (65.0, 102.0) | | 77.0 (63.0, 94.0) | | 89.0 (74.0, 107.0) | <0.001 |
| **Globulin** |  | |  | |  |  |
| min, median (IQR) | 28.0 (25.0, 30.0) | | 27.0 (24.0, 30.0) | | 28.0 (25.0, 30.0) | 0.064 |
| mean, median (IQR) | 31.2 (28.5, 34.0) | | 31.0 (28.3, 34.0) | | 31.5 (29.0, 34.1) | 0.064 |
| first, median (IQR) | 32.0 (29.0, 36.0) | | 31.0 (28.0, 35.0) | | 33.0 (30.0, 37.0) | <0.001 |
| **LDH** |  | |  | |  |  |
| max, median (IQR) | 253.0 (204.0, 344.0) | | 236.0 (193.0, 297.0) | | 297.5 (225.0, 385.0) | <0.001 |
| mean, median (IQR) | 209.7 (175.0, 260.1) | | 197.5 (168.0, 237.0) | | 232.6 (192.0, 289.5) | <0.001 |
| first, median (IQR) | 199.0 (164.0, 245.0) | | 195.0 (163.0, 244.0) | | 202.5 (168.0, 245.2) | 0.215 |
| **TBil** |  | |  | |  |  |
| max, median (IQR) | 16.9 (12.6, 24.6) | | 16.4 (12.6, 24.0) | | 17.6 (12.8, 25.6) | 0.138 |
| mean, median (IQR) | 12.1 (9.2, 16.9) | | 12.2 (9.3, 17.0) | | 12.0 (9.0, 16.8) | 0.317 |
| first, median (IQR) | 13.4 (9.9, 18.1) | | 13.2 (9.9, 17.8) | | 13.5 (9.8, 19.2) | 0.496 |
| **Total Protein** |  | |  | |  |  |
| min, median (IQR) | 60.0 (54.0, 65.0) | | 61.0 (56.0, 67.0) | | 58.0 (52.0, 63.0) | <0.001 |
| mean, median (IQR) | 66.8 (62.3, 71.0) | | 67.5 (63.5, 71.5) | | 65.2 (61.5, 69.8) | <0.001 |
| first, median (IQR) | 75.0 (69.0, 81.0) | | 74.0 (68.0, 80.0) | | 76.0 (70.0, 82.0) | <0.001 |

ALKP, alkaline phosphatase; ALT, alanine transaminase; AST, aspartate transaminase; GGT, gamma-glutamyl transferase; IQR, interquartile range; LDH, lactate dehydrogenase; TBil, total bilirubin

# Supplementary Table 3

**Supplementary Table 3.** The final hyperparameters of other five models

| **Hyperparameter** | **Final setting** |
| --- | --- |
| ****Deep Forest**** |  |
| 'max_layers' | 14 |
| 'n_estimators' | 1 |
| 'min_samples_split' | 8 |
| 'min_samples_leaf' | 8 |
| 'delta' | 8.609278613198186e-05 |
| ****Neural Network**** |  |
| 'units' | 37 |
| 'batch_size' | 38 |
| ****Decision Tree**** |  |
| 'max_depth' | 4 |
| 'criterion' | poisson |
| ****SVM**** |  |
| 'kernel' | rbf |
| ****Random Forest**** |  |
| criterion | poisson |
| n_estimators | 139 |
| min_samples_split | 9 |
| min_samples_leaf | 7 |

# Supplementary Table 4

**Supplementary Table 4.** Computational performance of machine models built without liver function markers.

| **Model** | **Accuracy** | **Precision** | **Recall** | **F1** | **AUC** | **Brier** |
| --- | --- | --- | --- | --- | --- | --- |
| Deep Forest | 0.855 | 0.798 | 0.845 | 0.821 | 0.906 | 0.117 |
| Neural Network | 0.846 | 0.787 | 0.833 | 0.809 | 0.913 | 0.153 |
| CatBoost | 0.832 | 0.735 | 0.893 | 0.806 | 0.922 | 0.113 |
| Decision Tree | 0.827 | 0.747 | 0.845 | 0.793 | 0.889 | 0.129 |
| SVM | 0.832 | 0.735 | 0.893 | 0.806 | 0.900 | 0.223 |
| Random Forest | 0.827 | 0.733 | 0.881 | 0.800 | 0.912 | 0.123 |

AUC, receiver operating characteristic curve; CatBoost, categorical boosting; SVM, support vector machine.

# Supplementary Table 5

**Supplementary Table 5.** The final hyperparameters of machine models built without liver function markers.

| **Hyperparameter** | **Final setting** |
| --- | --- |
| ****Deep Forest**** |  |
| 'max_layers' | 9 |
| 'n_estimators' | 2 |
| 'min_samples_split' | 2 |
| 'min_samples_leaf' | 4 |
| 'delta' | 1.1580217693365407e-05 |
| ****Neural Network**** |  |
| 'units' | 41 |
| 'batch_size' | 24 |
| ****CatBoost**** |  |
| loss_function | MultiRMSE |
| iterations | 107 |
| learning_rate | 0.008308762449388739 |
| depth | 5 |
| bagging_temperature | 1.2865874309633404e-05 |
| ****Decision Tree**** |  |
| 'max_depth' | 3 |
| 'criterion' | poisson |
| ****SVM**** |  |
| 'kernel' | poly |
| ****Random Forest**** |  |
| criterion | absolute_error |
| n_estimators | 103 |
| min_samples_split | 4 |
| min_samples_leaf | 6 |
